# CorSig: A General Framework for Estimating Statistical Significance of Correlation and Its Application to Gene Co-Expression Analysis

**DOI:** 10.1371/journal.pone.0077429

**Published:** 2013-10-23

**Authors:** Hong-Qiang Wang, Chung-Jui Tsai

**Affiliations:** 1 Intelligent Computing Lab, Hefei Institutes of Physical Science, Chinese Academy of Sciences, Hefei, China; 2 Department of Genetics, University of Georgia, Athens, Georgia, United States of America; 3 Warnell School of Forestry and Natural Resources, University of Georgia, Athens, Georgia, United States of America; Queen's University Belfast, United States of America

## Abstract

With the rapid increase of omics data, correlation analysis has become an indispensable tool for inferring meaningful associations from a large number of observations. Pearson correlation coefficient (PCC) and its variants are widely used for such purposes. However, it remains challenging to test whether an observed association is reliable both statistically and biologically. We present here a new method, CorSig, for statistical inference of correlation significance. CorSig is based on a biology-informed null hypothesis, i.e., testing whether the true PCC (*ρ*) between two variables is statistically larger than a user-specified PCC cutoff (*τ*), as opposed to the simple null hypothesis of *ρ* = 0 in existing methods, i.e., testing whether an association can be declared without a threshold. CorSig incorporates Fisher's Z transformation of the observed PCC (*r*), which facilitates use of standard techniques for *p*-value computation and multiple testing corrections. We compared CorSig against two methods: one uses a minimum PCC cutoff while the other (Zhu's procedure) controls correlation strength and statistical significance in two discrete steps. CorSig consistently outperformed these methods in various simulation data scenarios by balancing between false positives and false negatives. When tested on real-world *Populus* microarray data, CorSig effectively identified co-expressed genes in the flavonoid pathway, and discriminated between closely related gene family members for their differential association with flavonoid and lignin pathways. The *p*-values obtained by CorSig can be used as a stand-alone parameter for stratification of co-expressed genes according to their correlation strength in lieu of an arbitrary cutoff. CorSig requires one single tunable parameter, and can be readily extended to other correlation measures. Thus, CorSig should be useful for a wide range of applications, particularly for network analysis of high-dimensional genomic data.

**Software Availability:**

A web server for CorSig is provided at http://202.127.200.1:8080/probeWeb. R code for CorSig is freely available for non-commercial use at http://aspendb.uga.edu/downloads.

## Introduction

With the accumulation of large-scale data from various high-throughput technologies over the last decade, correlation network analysis has gained popularity for systems investigation of complex biological traits [1]–[3]. By studying the network topology of co-expressed genes under varying conditions, for instance, Carter et al. [4] showed that network connectivity is a reliable measure of condition-specific “essential genes” that may escape detection by conventional differential expression (DE) analysis. Gene network analysis has been exploited to understand regulatory mechanisms [5]–[9], including analysis of common promoter binding sites of co-expressed genes to infer co-regulation [10]. The interrogation of multi-omics profiling via network approaches is also of pivotal importance to the development of personalized medicine [11]–[13]. Central to these network inference approaches are correlation measures that seek to describe the relationship among variables. Commonly used correlation measures include Pearson correlation, Spearman correlation, mutual information and their variants, each with its own merits and limitations [14]–[23]. Pearson correlation and mutual information are well-suited for modeling linear and non-linear relationship, respectively, whereas conditional measures (e.g., partial Pearson correlation or conditional mutual information) are more effective for inferring non-static relationships [24].

A challenge in large-scale correlation analysis is to discern statistically significant relationships. Given two gene variables, their co-expression strength can be depicted by a correlation coefficient, such as Pearson correlation coefficient (PCC), calculated from the observed expression profiles. For a large number of genes, an arbitrary minimum PCC cutoff (MC) is often applied to identify biologically meaningful co-expression candidates, analogous to the use of an arbitrary fold-change cutoff in DE analysis. Although intuitive and simple, such a method does not control sampling errors and, therefore, is prone to false positives. Unlike the advances in multiple comparisons for DE analysis [25]–[29], rigorous statistical testing for correlation analysis remains under-developed [30], [31]

Several approaches are available for testing the significance of PCC (or the likes). One method is to test the null hypothesis that the true value of PCC (*ρ*) is equal to zero [32]. Thus, an association will be declared if the observed PCC is significantly different from zero. Another method infers significance by constructing a confidence interval for *ρ* at a given probability [32]. Other methods combining the above-mentioned approaches have also been proposed, such as the two-stage procedure of Zhu et al. [33], which attempts to control both statistical as well as biological significance. Statistical significance of the observed *r*'*s* at a given correlation threshold is first assessed by *p*-values calculated under the null hypothesis *ρ* = 0. A false discovery rate (FDR)-based confidence interval is then constructed to control false positives. In contrast to the simple MC methods, Zhu's method tends to be extremely stringent, due to the rigid two-stage workflow.

We argue that the simple null hypothesis is not applicable in genomics-level correlation analysis where the main challenge is to differentiate biologically significant correlations from those that occur by chance. To address this issue, we propose an alternative significance inference framework, i.e., testing if the following holds: 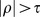
(1)where 0<*τ*<1 is a given threshold for an observed *r* (both positive and negative correlations can be considered using the absolute value of *r*). Eq.(1) represents an improvement over Zhu's two-stage procedure in that we seek to simultaneously control biological and random errors in an integrative, rather than discrete, manner, and that we infer statistical significance under a more biologically relevant context of Eq.(1) rather than the simple null hypothesis *ρ* = 0. We devised a statistical procedure, based on Fisher's Z-transformation of PCC, that facilitates the use of standard statistical techniques and multiple testing corrections for reliable identification of co-expressed genes. The proposed method, CorSig, can be extended to other correlation measures and should be applicable to other genomics studies, such as protein-protein interaction, metabolomics and genetic linkage analyses.

## Methods

### Relevant Probability Distribution Theories

An intuitive option for solving the inference problem of 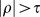 is to use the probability distribution of sample PCC *r*, denoted by D(*r*). However, D(*r*) is not well understood. Some properties of the distribution have been reported under certain data scenarios. For two uncorrelated variables that follow a bivariate normal distribution, for example, *r* approximately follows a *t*-distribution with a degree of freedom *n*-2 (*n* is the sample size) [32], [34]. Depending on the data, D(*r*) can be influenced by the true PCC *ρ* and the sample size *n*. For dataset with *ρ* = 0, *r* is symmetrically distributed around 0, and therefore, is an unbiased estimator of *ρ*. When *ρ*>0 or *ρ*<0, however, the distribution of *r* will be skewed toward the negative or the positive side, respectively. Finally, *n* can influence D(*r*), as the larger the *n*, the smaller the standard deviation of *r*.

Fisher [35] developed a variance-stabilizing transformation to convert *r* values into weighted *z* scores. This provides a convenient means by which to draw inference on *ρ* when it is different from zero. Without loss of generality, we write the Fisher's Z-transformation as: 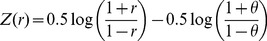
(2)where 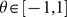 is a constant and can be conveniently set as *θ* = *τ* (see explanation in the next section). In contrast to our limited understanding of D(*r*), extensive studies have been conducted on the properties of *Z* [36]–[38]. Given *ρ*, *Z* is approximately normally distributed with mean calculated from *ρ* via Eq.(2) and standard deviation (SD), defined as: 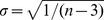
(3)


In the following section, we describe the application of the Fisher transformation to the proposed statistical inference about 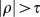.

### The CorSig Framework of Identifying Reliable Co-expressions and Its Statistical Solution

For the presentation of the proposed framework, we assume a gene expression data set containing the expression levels of *m* genes in *n* observations (samples), denoted as a matrix of *m* rows and *n* columns: 
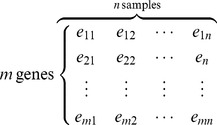
(4)


The expression vector of each gene can be represented as 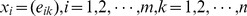. So, the observed PCC between two genes, *i* and *j*, can be calculated as 
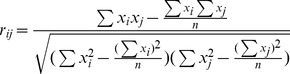
(5)


We aim to determine whether the true PCC *ρ* between genes *i* and *j* is significantly larger than a given threshold *τ* by the observed value *r_ij_*. More specifically, we estimate the *p*-value of *r_ij_* under 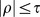.

To solve the significance problem, we assume the following null hypothesis *H*_0_: 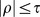 against the alternative hypothesis *H*_1_: 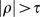. In standard statistical theory, the null hypothesis *H*_0_ is termed composite null hypothesis in that it specifies an interval of values for *ρ* rather than a fixed value as seen in the simple null hypothesis (*ρ* = 0). Unlike the single-value null hypothesis test that has been relatively well studied, there is no specific procedure in standard statistical theory for the composite null hypothesis test [32], [39]. It has been suggested that an approximation can be obtained by constructing a likelihood ratio test of *H*_0_ versus *H*_1_ and then applying the asymptotic distribution theory [40]. Here, we consider the probability of 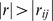, where *r* represents sample PCC's under *H*_0_. Let *p* denote the *p*-value of *r_ij_*, *p* can be estimated as the conditional probability Pr(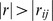
|*H*_0_). As *H*_0_ represents an interval of *ρ*, it is not possible to directly calculate the conditional probability using standard statistical theory. To bypass this problem, we choose an element of *H*_0_ which is the most difficult to reject as an upper bound for the conditional probability. For positive *r_ij_*'s, the most stringent *H*_0_ to reject is *ρ* = *τ*, while for negative *r_ij_*'s, it is *ρ* = −*τ*. Thus, the resulting inference procedure can be formulated as 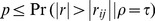
(6)


It should be noted that either *ρ* = *τ* or the absolute value of *ρ* = *τ* will produce the same *p* estimate mathematically. Using Eq.(6), therefore, we can estimate the upper bound (conservative) *p*-value (hereafter, referred to as *p*-value), i.e., 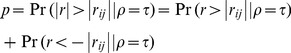
(7)


Because the distribution of *r* under *ρ* = *τ* is unknown, Eq.(7) can not be immediately calculated. Considering the probability distribution theories discussed in the previous section, we turned to Fisher's Z-transformation of *r* under *ρ* = *τ* for estimating *p*. We denote the Fisher's Z-transformation of *ρ*, *r_ij_* and *τ* by *z_ρ_*, *z_ij_* and*z*_r_, respectively, which can be calculated by Eq.(2). Because Fisher's Z-transformation is an increasing function, Eq.(7) can be equivalently rewritten as 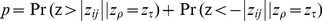
(8)where *z* represents the Fisher's Z-transformation of *r* by Eq.(2) and follows a normal distribution 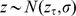 as described above. More intuitively, we set *θ* = *τ* in Eq.(2) to have 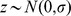, *i*.*e*., *z* is symmetric around zero. Therefore, the *p*-value of *r_ij_* can be estimated by Eq. (8) as the sum of the upper-tail and lower-tail probabilities of a normal distribution centered at zero. We note that this *p*-value is a conservative estimation as an upper bound conditional probability.

### Simulation Data Generation

We applied the following procedures to generate various types of simulation data for model evaluation. 1) Sampling *n* numbers from the standard normal distribution *N*(0,1) as the target variable *t*, and 2) randomly generating *G* = 1000 variables 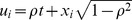, *i* = 1,2,…,*G*, where 0<*ρ*<1 is a constant, representing the population PCC between *μ_i_* and *t*, and *x_i_* is a standard normal random variable uncorrelated with *t*. The procedure makes sure that *t* and *μ_i_* are correlated with a population PCC of *ρ*.

For Simulation Data I, each of the *G* variables was generated by using a *ρ* uniformly sampled from a range (0,1). By varying *n* among 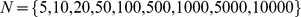, we generated nine such data sets. For each data set, we aimed to identify the correlated variables with *ρ*>*τ*, 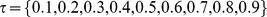.

For Simulation Data II, each of the *G* variables was generated by using a fixed *ρ*, and by varying *n* among *N* and *ρ* among 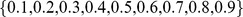 respectively, to produce 81 (9×9) data sets. The Simulation Data II were designed to observe how significantly an observed SD (*σ*) deviates from the theoretical values, and how *n* and *ρ* influence the results.

For Simulation Data III, 900 of the *G* variables were generated by using a *ρ* uniformly sampled from (0,*τ*) and the remaining 100 by using a *ρ* uniformly sampled from a sub-range (*τ*,1). At the same time, noise was added to the data by making *x_i_* correlated with *t* at a random PCC around 0.2. The introduced noise is expected to inflate *r*, possibly leading to more false positives (FP) than false negatives (FN). Similar to Simulation Data II, 9×9 such data sets were generated by varying *n* and *τ* for algorithm evaluation. Compared with Simulation Data I and II, Simulation Data III is more challenging due to the complex structure and the added noise.

Simulation Data IV were similarly generated as Simulation Data III, except using a non-normal Chi-square distribution with degree of freedom set at 5 and a non-centrality of 1. As with other cases, 9×9 data sets were obtained by varying *n* and *τ* for different data scenarios. Compared to the other data types, Simulation Data IV has a long tailed distribution, which is expected to produce spurious correlation coefficients between unrelated variables, thus complicating the analysis.

## Results

### The Properties of the CorSig *p*-value on Normal Data

**Figure 1:**
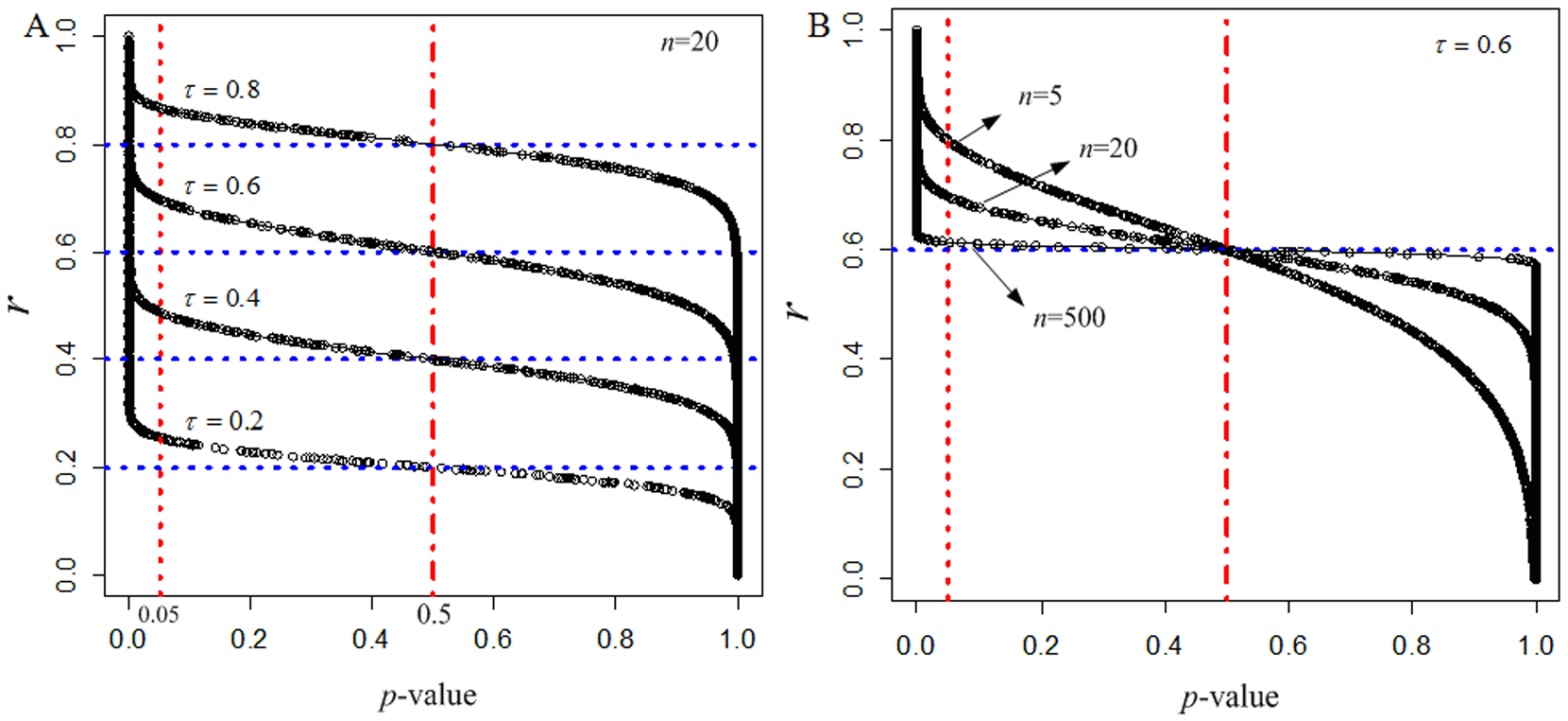
Relationship of *p*-values and observed *r*'*s*. A is for four cases of *n* = 20; B is for three cases of *τ* = 0.6.

We first evaluated the proposed method using Simulation Data I. After obtaining the observed *r*'*s* for all pairwise comparisons of variables, *p*-values were calculated using the theoretical SDs from Eq.(3) for each of the nine data scenarios. Fig. 1A shows the relationship between the *p*-value and the observed *r* for four scenarios with different *τ* from the *n* = 20 data sets. The calculated *p*-values are inversely proportional to the observed *r*'s for any *τ*, as one may predict. It also shows that the *p*-value at *τ* is 0.5 for all *τ* scenarios. This is due to the asymptotic normality of the Fisher transformation of the observed *r*'s. When *ρ* = *τ*, there exists a probability of 0.5 for an observed PCC being no larger than the true PCC *ρ* = *τ*.

Fig. 1B revealed a strong influence of *n* on the *r* vs. *p*-value relationship curves. In particular, as *n* increases, the *p*-values become increasingly biased toward the extremes (0 and 1), due to the gradually decreasing deviation of an observed Z(*r*) from its population *Z*(*ρ*). In light of the relationship of SD and *n* depicted in Eq.(3), this suggests that the value of SD can greatly influence the calculation of *p*-values in the proposed method. Using a *p*-value cutoff of 0.05, we identified the variables that were significantly correlated with the target variable *t* under various data scenarios, as shown in Table 1A. The number of variables declared significant was found to increase as *n* increased and approached the corresponding true numbers when *n* = 500 or higher, irrespective of the *τ*. This is consistent with the limitation of Eq.(3) in that SD can be reliably estimated as a function of the sample size *n* only when *n* is sufficiently large. For a small or moderate *n*, Eq.(3) does not hold [9].

**Table 1:** Numbers of variables identified by CorSig with a *p*-value cutoff of 0.05, using a theoretical value of SD (A), or a fitted value of SD (B), using Simulation Data I.

A. Theoretical SD										
	True#	n = 5	n = 10	n = 20	n = 50	n = 100	n = 500	n = 1000	n = 5000	n = 10000
*τ* = 0.2	807	129	342	465	567	628	734	758	791	793
*τ* = 0.4	599	80	244	340	389	439	531	557	583	591
*τ* = 0.6	395	49	154	217	260	286	344	366	385	391
*τ* = 0.8	198	21	70	103	123	132	171	185	192	197
B. Fitted SD										
	True#	n = 5	n = 10	n = 20	n = 50	n = 100	n = 500	n = 1000	n = 5000	n = 10000
*τ* = 0.2	807	601	719	749	757	770	789	796	804	806
*τ* = 0.4	599	361	466	520	521	536	578	580	596	598
*τ* = 0.6	395	192	281	316	317	321	370	379	390	395
*τ* = 0.8	198	78	122	141	155	156	180	191	197	199

### Influence of the SD

**Figure 2:**
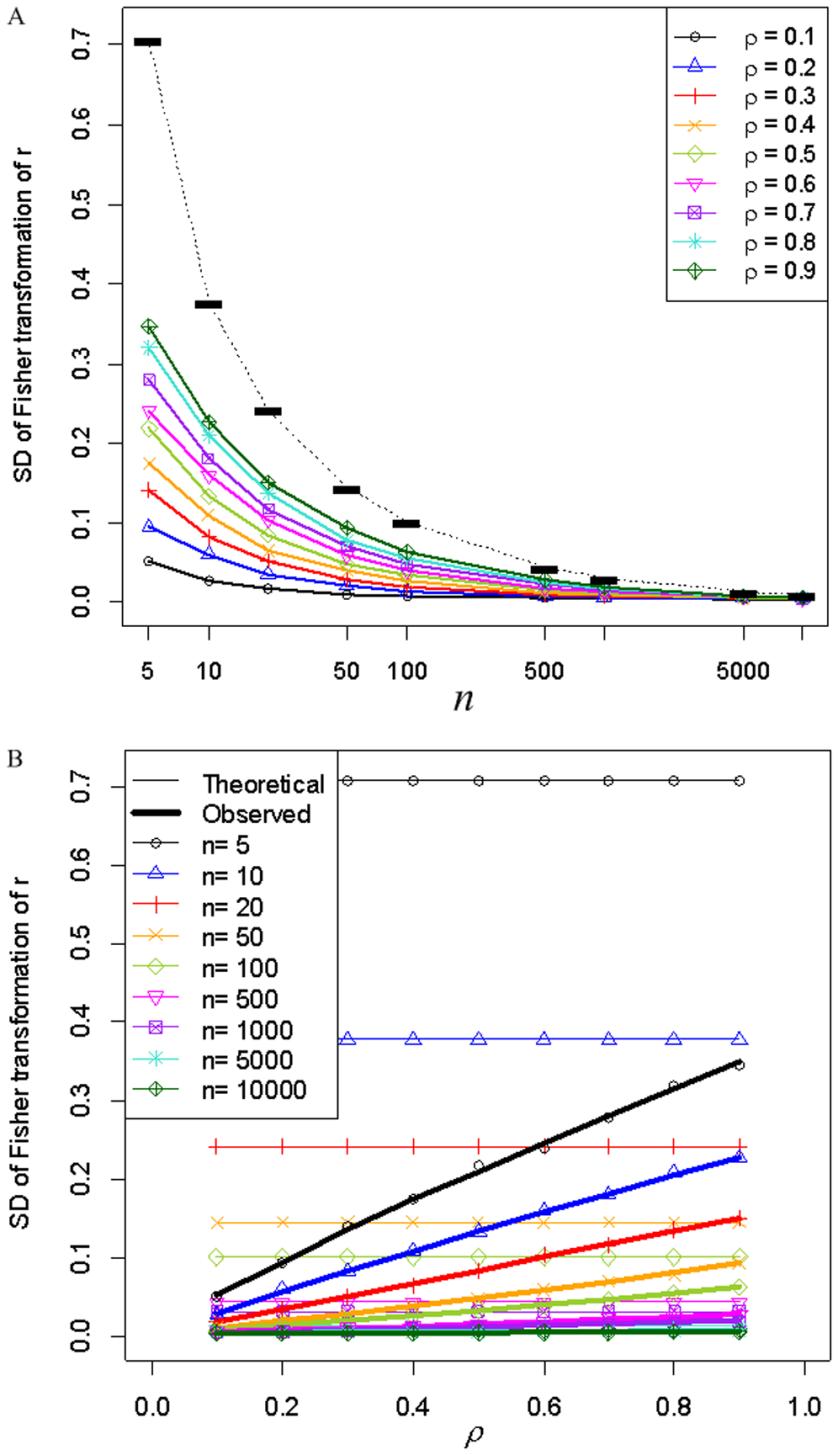
The observed SD varied according to *n* and *ρ*, in sharp contrast to the invariable theoretical SD. A. The influence of *n* across nine different *ρ* values. The nine corresponding theoretical SDs are connected by dotted lines. B. The influence of *ρ* on the observed SDs (thick lines) across nine *n* levels. The corresponding theoretical SDs are shown in thin lines.

We next investigated the empirical distribution of SD, especially when *n* is small, using Simulation Data II. In each data scenario, the observed *r*'s of the 1000 variables share a common *ρ*. We therefore Fisher-transformed the *r*'s and calculated the SDs for the fixed *ρ*. As shown in Fig. 2, the observed SDs varied with changing *n* and *ρ*, whereas the corresponding theoretical values obtained by Eq.(3) did not. Specifically, the observed SDs were always lower than the theoretical values, approaching the theoretical values only when *n* is very large (e.g, >5000) (Fig. 2A). This translates into an underestimate of significantly correlated variables in data scenarios with a small *n*, as obtained for Simulation Data I (Table 1A). When *n* is small, the SD is greatly influenced by both *n* and *ρ*, as shown in Fig. 2B. This indicates that the theoretical SD is not reliable enough to be adopted in practice.

In light of the observations above, we used the observed SDs for Simulation Data II (Fig. 2) to train a regression model for SD estimation based on *n* and *ρ*, using the LOWESS algorithm [41] in *R* package STATS. Empirical testing of various smoothing parameters and polynomial degrees produced essentially the same model, therefore, the default settings (0.75 and 2, respectively), were used. We revisited the Simulation Data I and used the learned model to obtain the LOWESS-fitted SDs for each data scenario. The fitted SD values were then used to re-calculate the *p*-values for each of the nine data scenarios. As shown in Table 1B, the numbers of variables predicted as significant were much closer to the true values when compared to those shown in Table 1A, especially for data sets with *n* ≤100. The results suggest that an improved SD estimation can enhance the performance of the proposed significance inference.

**Figure 3:**
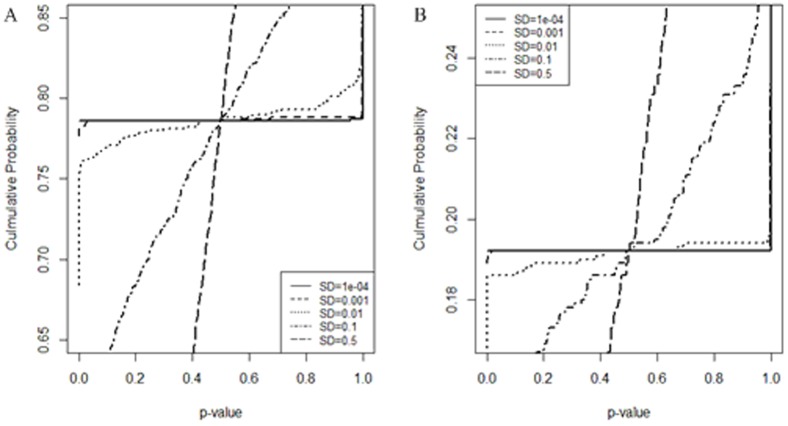
Cumulative probability distribution of the *p*-values calculated using different values of SD. A is for the *n* = 5 & *τ* = 0.2 data scenario and B the *n* = 5 & *τ* = 0.8 data scenario.

To further evaluate the influence of SD on the proposed CorSig, we repeatedly changed the SD and re-calculated the *p*-values using the Simulation Data I. The cumulative probability curves of the resultant *p*-values under different SDs are shown in Fig. 3 for two data scenarios. In both cases, as SD decreased, the *p*-value distribution became increasingly distorted toward the two extremes. In particular, when SD≤0.001, the *p*-values were either close to 1 (for variables with 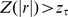) or 0 (for variables with 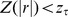). This pattern resembles the outcome of simply comparing the observed *r* with *τ* using the MC method. In other words, the MC method can be regarded as an extreme version of CorSig with a very small SD. Taken together, the results suggest that SD can be treated as a tunable parameter to improve significance estimation.

### Evaluation on Normal Data with Complex Structure and Added Noise

We compared CorSig with fitted SDs against the simple MC method as well as Zhu's procedure on the Simulation Data III, designed to contain complex structure and data noise. Significance was declared by a PCC cutoff (*τ*) alone in MC, along with an arbitrary *p*-value cutoff of 0.05 in CorSig, or following the default parameters in Zhu's [33]. As expected, the MC method detected a large number of FP, especially for data sets with a small *n*, but was generally effective against FN, with essentially no FN from data sets with a large *n* (Table 2). In comparison, CorSig had considerably fewer FP, but sometimes at the expense of more FN (e.g., when *n* is small and *τ* is large). By contrast, Zhu's procedure led to more FN than FP in almost all cases (Table 2). Although the results of CorSig were intermediate between the two extremes (MC and Zhu's), its overall bias toward more FP than FN for data sets with small *n* and *τ* highlighted the need for additional tuning.

**Table 2:** FP and FN discoveries* by MC, CorSig and Zhu's procedures for different Simulation Data III scenarios.

	n = 5	n = 10	n = 20	n = 50	n = 100	n = 500	n = 1000	n = 5000	n = 10000
MC									
*τ* = 0.1	725/11	628/6	534/9	448/5	318/2	147/1	118/0	43/0	35/0
*τ* = 0.2	533/25	430/7	303/9	159/6	183/1	76/0	48/0	23/0	18/0
*τ* = 0.3	392/31	287/10	216/4	223/2	92/1	48/1	34/0	23/0	17/0
*τ* = 0.4	273/26	128/36	75/20	67/4	91/1	28/1	38/2	18/1	12/0
*τ* = 0.5	225/9	45/49	66/7	12/20	27/6	35/1	17/0	11/0	4/0
*τ* = 0.6	156/78	27/40	95/8	113/1	24/5	11/5	25/0	7/0	5/0
*τ* = 0.7	71/47	72/5	15/18	72/1	15/8	9/0	19/0	7/0	6/0
*τ* = 0.8	114/12	55/11	5/32	16/11	16/7	4/5	5/2	2/0	2/0
*τ* = 0.9	24/31	21/9	7/17	41/0	20/3	9/0	7/0	7/0	5/0
CorSig (fitted SD)									
*τ* = 0.1	575/17	506/8	434/12	365/5	261/2	113/1	76/0	18/0	1/0
*τ* = 0.2	327/44	255/13	186/11	87/8	137/3	58/1	23/1	11/0	3/0
*τ* = 0.3	212/53	110/19	105/9	157/4	56/5	32/1	12/3	12/0	0/0
*τ* = 0.4	116/52	47/50	17/30	18/8	52/2	18/3	15/4	7/3	0/1
*τ* = 0.5	69/29	12/70	10/21	0/29	11/13	19/2	7/2	5/0	1/1
*τ* = 0.6	54/93	6/35	19/17	44/7	7/14	5/8	8/3	2/3	0/5
*τ* = 0.7	20/68	14/20	1/37	23/9	1/18	2/3	3/3	0/1	0/1
*τ* = 0.8	16/44	7/46	7/24	3/33	1/18	0/8	0/7	1/0	0/0
*τ* = 0.9	2/63	1/49	2/38	12/5	4/9	6/1	0/4	0/0	0/0
Zhu's procedure									
*τ* = 0.1	0/100Δ	1/79	0/71	0/49	0/31	0/17	0/3	0/4	0/4
*τ* = 0.2	0/100Δ	0/99	0/84	0/82	0/46	0/22	0/26	0/20	2/18
*τ* = 0.3	0/100Δ	0/79	0/52	1/21	1/30	2/9	2/9	2/1	2/2
*τ* = 0.4	0/100Δ	0/100Δ	0/91	0/68	0/42	0/29	0/22	0/19	0/21
*τ* = 0.5	0/100Δ	0/100Δ	0/74	0/62	0/38	0/16	0/13	0/8	0/8
*τ* = 0.6	0/100Δ	0/100Δ	0/75	0/35	0/42	0/27	0/14	0/16	0/15
*τ* = 0.7	0/95	0/72	0/67	2/24	2/32	2/17	2/6	2/5	3/4
*τ* = 0.8	0/100Δ	0/85	0/82	0/58	0/45	0/24	0/15	0/6	0/4
*τ* = 0.9	0/100Δ	0/93	0/78	0/29	0/34	0/18	0/19	0/13	0/13

*Data are presented as FP/FN;

Δ Reaching the maximum of FN.

**Figure 4:**
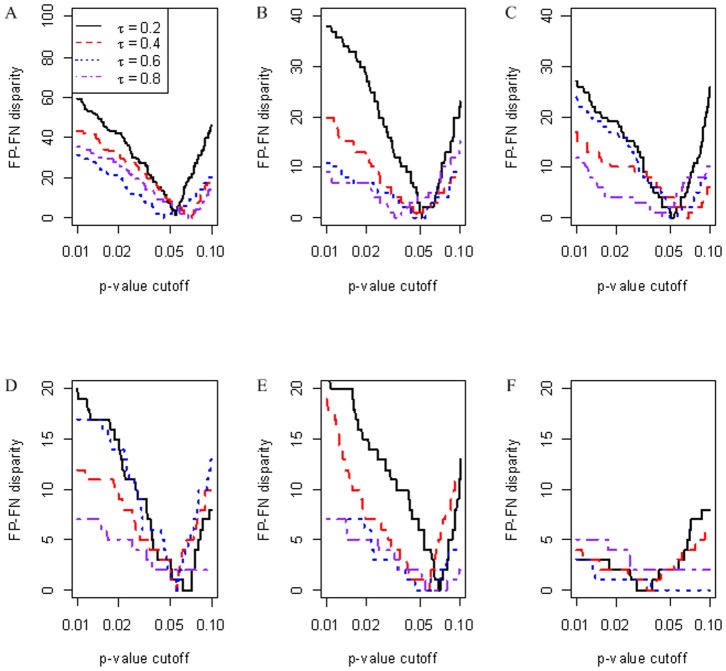
Changes of FP-FN disparity at *p*-value cutoffs between 0.01 and 0.1 for Simulation Data III. A–F are for scenarios of *τ* = 0.2,0.4,0.6,0.8 with n = 5 (A), 10 (B), 20 (C), 50 (D), 100 (E) and 500 (F), respectively. Note the different y-axis scales.

Based on the idea that SD may be a tunable parameter and that too small or too large SDs tend to produce very extreme *p*-value estimations (Fig. 3), we reasoned that a proper SD may help deal with complex or noisy data like the Simulation Data III. We tuned the values of SD to re-calculate the *p*-values for Simulation Data III. Based on the resultant *p*-values, the FP-FN disparities at different significance cutoffs are summarized in Fig. 4 for scenarios of *τ* = 0.2, 0.4, 0.6 and 0.8 with a small or moderate *n* = 5 (A), 10 (B), 20 (C), 50 (D), 100 (E) and 500 (F). The disparities reached the minimum in all cases at a *p*-value cutoff between 0.01 and 0.1. The chosen SD values and the resultant FP and FN at a *p*-value cutoff of 0.05 for representative data sets are shown in Table 3. The results provided experimental support for the suitability of SD as a tunable parameter to obtain a more balanced significance measure than those by MC, Zhu's procedure or with the fitted SD (Table 2). Because SDs are inversely proportional to *n* (Table 3), users can choose values of SD according to the experimental sample size. We recommend an SD from the range of [0.2,0.4] for data sets with small sample sizes (*n*≤5) and an SD of [0.01,0.15] for moderate sample sizes (5<*n*≤500). In general, the performance of CorSig on large data sets (*n*≥500) appeared less sensitive to SD. In such cases, an SD can be chosen by the LOWESS regression presented above or from the range of [0.001, 0.04].

**Table 3:** Results by CorSig using an adjusted SD on Simulation Data III.

	FP/FN	SD	FP/FN	SD	FP/FN	SD	FP/FN	SD
	n = 5		n = 10		n = 20		n = 50	
*τ* = 0.2	95/73	0.40	35/37	0.25	20/22	0.15	16/18	0.08
*τ* = 0.4	54/62	0.40	50/49	0.10	25/29	0.05	13/14	0.05
*τ* = 0.6	90/88	0.20	24/24	0.08	17/17	0.10	15/16	0.10
*τ* = 0.8	30/37	0.25	29/25	0.08	19/17	0.08	10/12	0.02
	n = 100		n = 500		n = 1000		n = 5000	
*τ* = 0.2	9/15	0.08	6/4	0.04	7/5	0.025	3/2	0.012
*τ* = 0.4	11/12	0.08	6/4	0.02	5/4	0.025	4/3	0.012
*τ* = 0.6	11/11	0.03	7/7	0.01	4/3	0.025	2/1	0.005
*τ* = 0.8	7/9	0.02	4/5	0.001	2/2	0.005	1/0	0.001

### Evaluation on Non-normal Data

We next evaluated CorSig on Simulation Data IV with non-normal distribution. For these data sets, spuriously large sample correlation coefficients are expected due to the long tailed data distribution, and more outliers will be introduced with larger sample sizes and larger population correlation. This was indeed the case, based on the overall higher false positive rates (FPR) obtained by the three methods (Table 4). The MC method showed a severe bias towards high FPR across all data scenarios, especially when sample sizes are large. Zhu's method, on the other hand, showed two distinct patterns of imbalanced discoveries, biasing toward high false negative rates (FNR) when samples sizes were small but exhibiting high FPR with increasing sample sizes. In comparison, CorSig with SD = 0.2 or 0.5 obtained more balanced FPR and FNR for almost all the data scenarios. It was also observed that larger SDs were needed to deal with more outliers in the cases of larger sample sizes and larger population correlations. Together, these results confirm that CorSig is more effective than the previous methods for detecting significant correlations even for the non-normal data.

**Table 4:** FPR(%)/FNR(%) by MC, CorSig and Zhu's procedures for different Simulation Data IV scenarios.

*τ*\n	5	10	20	50	100	500	10^3^	5000	10^4^	5	10	20	50	100	500	10^3^	5000	10^4^
MC										Zhu's procedure								
0.1	82/10	74/6	72/3	66/1	70/0	73/0	73/0	74/0	73/0	0/97	0/53	0/13	0/7	11/0	46/0	55/0	66/0	67/0
0.2	64/6	59/2	51/2	66/0	70/0	72/0	73/0	73/0	72/0	0/93	0/53	0/42	22/14	35/11	61/7	66/8	70/7	70/7
0.3	52/18	49/7	57/6	69/0	70/1	75/0	74/0	76/0	76/0	0/97	0/53	**9/1**	41/0	47/0	65/0	68/0	71/0	73/0
0.4	49/10	54/6	62/3	70/1	68/0	72/0	70/0	73/0	73/0	0/88	4/23	31/7	50/4	54/3	65/1	65/1	70/1	71/1
0.5	37/12	47/4	47/2	56/0	69/0	67/0	65/0	67/0	67/0	0/82	**9/15**	**14/2**	36/1	55/1	62/1	61/1	65/1	66/1
0.6	53/4	57/2	41/8	71/0	66/1	64/0	67/0	69/0	69/0	0/58	23/4	**7/3**	58/2	56/1	60/2	64/2	67/2	68/2
0.7	28/11	33/11	56/0	54/1	66/0	62/0	64/0	65/0	65/0	0/82	0/31	37/0	40/0	58/0	57/0	60/0	63/0	64/0
0.8	13/31	41/2	36/3	61/0	54/1	60/0	60/0	57/0	57/0	0/97	12/2	**13/2**	48/1	44/1	56/1	56/1	56/1	57/1
0.9	21/13	15/23	40/1	57/0	47/0	51/0	49/0	48/0	48/0	0/84	0/64	20/5	46/3	36/4	48/3	46/4	47/4	47/3
CorSig (SD = 0.2)										CorSig (SD = 0.5)								
0.1	**34/39**	**11/16**	**9/6**	**3/5**	**2/2**	**1/2**	**0/0**	**0/0**	**0/0**	7/62	0/38	0/16	0/26	0/19	0/18	0/17	0/17	0/18
0.2	**24/21**	**14/11**	**8/6**	22/1	21/0	27/0	26/0	26/0	26/0	4/33	0/27	0/29	**0/18**	**0/12**	**0/12**	**0/11**	**0/11**	**0/12**
0.3	**19/33**	**17/16**	**28/10**	38/4	32/1	41/0	41/0	42/0	42/0	4/54	0/40	0/26	**1/16**	**0/19**	**0/11**	**0/11**	**0/11**	**0/11**
0.4	**23/21**	**32/14**	43/7	48/1	45/0	45/0	44/0	47/0	48/0	7/42	7/21	**15/14**	**1/2**	**2/1**	**2/0**	**0/1**	**3/0**	**3/0**
0.5	**21/24**	32/13	27/6	35/4	49/2	47/0	44/0	47/0	47/0	6/40	12/24	3/14	**3/12**	**16/3**	**1/0**	**6/0**	**9/0**	**9/0**
0.6	38/13	44/7	21/16	56/1	48/3	45/0	49/0	52/0	52/0	**20/22**	**26/18**	0/34	**32/2**	**15/6**	**7/0**	**13/0**	**17/0**	**16/0**
0.7	**15/22**	**18/23**	44/5	39/4	51/0	45/0	47/0	47/0	48/0	4/36	2/42	**26/11**	**10/6**	**25/3**	**15/0**	**17/0**	**19/0**	**19/0**
0.8	**6/45**	31/09	21/9	47/1	37/2	47/0	45/0	42/0	42/0	1/70	**15/15**	**2/13**	**25/6**	**12/6**	**21/0**	**20/0**	**17/0**	**17/0**
0.9	**15/23**	**6/31**	27/7	43/1	31/3	36/0	33/0	32/0	32/0	6/36	0/58	**12/16**	**23/5**	**12/5**	**16/0**	**14/0**	**13/0**	**13/0**

Note: Best results are indicated in bold.

### Evaluation on Real-world Gene Expression Data

We applied CorSig to a *Populus* Affymetrix microarray data set encompassing 20 experimental conditions that examined gene expression changes of leaves or roots in response to various perturbations (each with 2 biological replicates) [42]. Raw hybridization signals were processed using the R package affyPLM, and *m* = 5463 probes that passed quality control (QC) filtering (raw intensities ≥100 in all 40 samples) were obtained for co-expression analysis.

**Figure 5:**
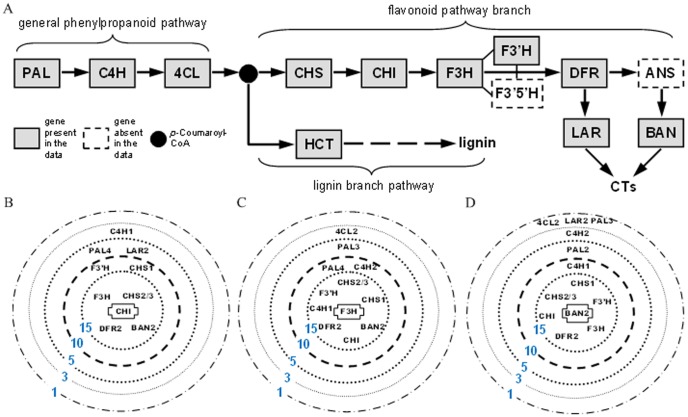
CEL stratifications of co-expressed genes in *Populus* microarray data analysis. A is the simplified flavonoid pathway and B–D are stratifications of co-expressed genes by CEL for seed genes, CHI (B), F3H (C) and BAN2 (D). In B–D, only flavonoid gene names are given, and 5-CEL (CEL = 1, 3, 5, 10 and 15) stratifications are shown for each seed gene.

We considered the relatively well-studied flavonoid biosynthetic pathway that gives rise to a suite of secondary metabolites, including condensed tannins (CTs), that are abundant in *Populus* and serve important defense functions [43]. Fig. 5A shows the simplified pathway. This pathway is known to be under transcriptional regulation [44], and co-expression of the *Populus* genes has been previously reported [43]. Of the nine enzymatic steps depicted in the flavonoid pathway branch, seven gene families (nine isoforms), including CHS, CHI, F3H, F3'H, DFR, LAR and BAN, were represented in the data, in addition to three upstream steps (PAL, 4CL and C4H, seven isoforms) of the general phenylpropanoid pathway. We compared CorSig, MC and Zhu's for identifying genes that were significantly co-expressed with each of the flavonoid genes, using a range of *τ* (0.2 to 0.8) [33]. As Zhu's method controls FDR [45], the same multiple testing correction was also applied to the *p*-values obtained by CorSig for a fair comparison. In both cases, FDR cutoff was set at 0.01.

**Figure 6:**
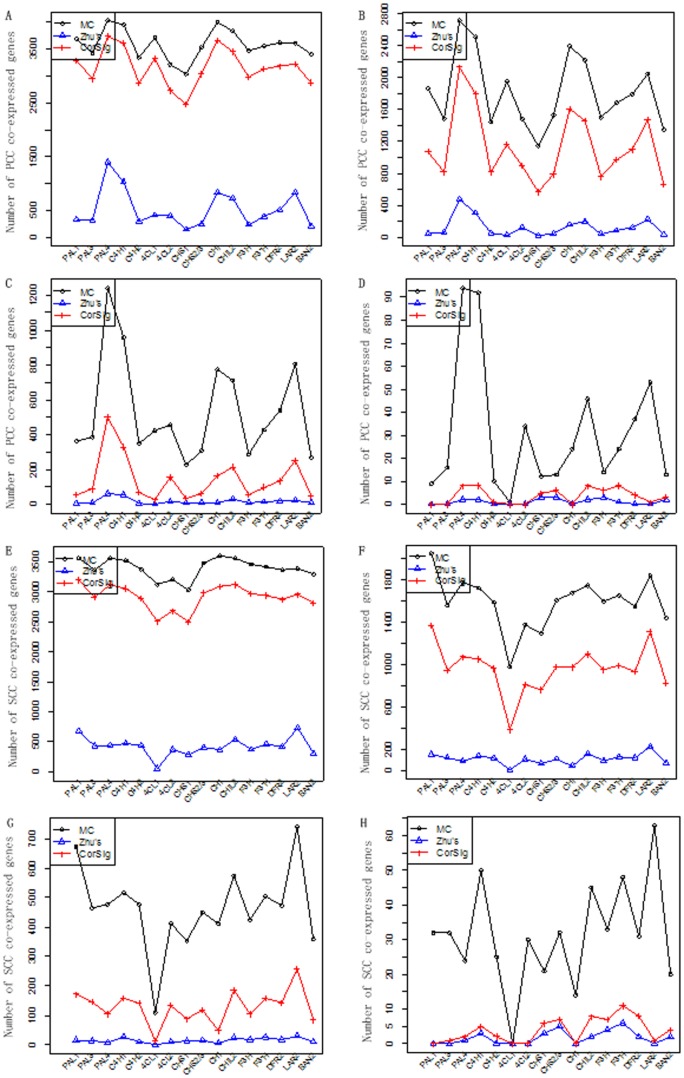
Identification of genes significantly co-expressed with the *Populus* phenylpropanoid-flavonoid pathway genes (x-axes) by the three methods. The analysis was performed with Pearson correlation (A–D) or Spearman correlation (E–H) using *τ* = 0.2 (A,E), 0.4 (B,F), 0.6 (C,G), or 0.8 (D,H).

Consistent with the findings from the simulation data sets, the numbers of significantly correlated genes identified by CorSig were much lower than those from MC but higher than those of Zhu's in all cases examined (Fig. 6A–D). Similar results were obtained using the Spearman correlation coefficient (SCC) in place of PCC (Fig. 6E–H). Examination of the results using *τ* = 0.6 as an arbitrary cutoff [33] showed that Zhu's method missed several co-regulating flavonoid genes. For example, only three flavonoid pathway genes were found to exhibit significant co-expression with CHI, while CorSig identified all eight of them, plus two upstream (PAL4 and C4H1) genes (Fig. 5B). As another example, the two 4CL isoforms present in our dataset, 4CL1 and 4CL2, are known to be differentially associated with lignin and flavonoid biosynthesis, respectively [46], [47]. For the flavonoid-related 4CL2, Zhu's procedure called 15 genes with significant co-expression, but none associated with the phenylpropanoid-flavonoid pathway. CorSig identified six genes in this pathway (PAL3, C4H2, CHS1, CHIL2, DFR2, and F3'H) that showed significant correlation with 4CL2. No significant co-expression was found for the lignin-specific 4CL1 by Zhu's, whereas CorSig detected eight such genes, including PAL4 and C4H1 and HCT1 that have been previously shown to exhibit preferential expression in lignifying tissues by multiple research groups [43], [48], [49]. The low number of genes co-expressed with 4CL1 was not surprising, since lignifying tissues were not well-represented in the *Populus* dataset used. Nevertheless, the results demonstrate that CorSig-assisted correlation analysis is effective for identifying biologically meaningful co-expression patterns.

Another application of the proposed significance testing is that the *p*-values obtained from CorSig provide an alternative measure to PCC for co-expression analysis between the seed and the tested genes. For each seed gene, we assigned the associated genes into six co-expression levels (CEL), {0,1,3,5,10,15}, based on the negative log10 transformed *p*-values. This in effect stratified genes of interest according to the statistical support of their co-expression with the seed, which reflects the PCC strength. The results can be visualized as a concentric stratification graph centered around the seed, with co-expressed genes arranged in decreasing order of statistical support from inner to outer rings (Fig. 5B–D). As shown for three seed genes from the flavonoid pathway, most of the other phenylpropanoid-flavonoid pathway members were found to be co-expressed at the highest CELs (15 and 10). The data also revealed that the CELs of the co-expressed genes corresponded reversely to their relative distances to the corresponding seed in the pathway (Fig. 5A), suggesting different regulation between early and late pathway genes.

## Discussion

In this paper, we have defined a new type of statistical inference problem for correlation analysis, and proposed a method (CorSig) to compute *p*-values for all observed correlations at a user-defined co-expression threshold (*τ*). CorSig requires only one adjustable parameter, SD (*σ*). For a sufficiently large sample size *n*, *σ* is a function of *n* and can be calculated conceptually. However, for a small or moderate *n*, *σ* is influenced by both *n* and the true PCC (*ρ*) according to simulation analysis. Based on this observation, a LOWESS regression model was used to compute *σ* according to *n* and *ρ*. The model was shown to be effective for normal data sets with a low level of noise (represented by Simulation Data I) or for complex data sets with large sample sizes (represented by Simulation Data III).

For complex data with small sample sizes or a non-normal distribution, a true *σ* value is not necessary for the *p*-value estimation, due to the asymptotic property of Fisher's *Z* transformation. Empirical testing with Simulation Data III and IV showed that in such cases, a larger *σ* value can be used to obtain a more balanced result between FP and FN. Choosing a proper *σ* may be challenging for complex data in practice, which is beyond the scope of the present investigation and will be addressed in the future. In practice, it is highly recommended that genomic data be pre-processed and normalized by appropriate QC measures to remove data noise and to reduce the occurrence of spurious correlation coefficients that can affect data inference. In our experience, skewed data distribution can often be alleviated after QC filtering. By examining data distribution patterns, the researchers will be able to select an appropriate SD estimation means: a fitted SD typically works well for normal data, while a larger SD is more appropriate for complex or non-normal data (Table 4).

In theory, TN should have a small observed 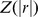 while TP should have a large 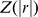. Accurate significant measure should ideally minimize both FP and FN. In practice, however, biological data are often influenced by non-experimental or non-controlled factors in such a way that TN could have an *r* as large as that of TP and vice versa. In these situations, high rates of FP and FN or an unbalanced FP/FN discovery would result. We showed that the use of an arbitrary correlation cutoff tends to produce a large number of TP due to the lack of significance control, while Zhu's method tends to be extremely conservative. The proposed CorSig provides an optimal solution by maintaining the FP and FN balance for Simulation Data III with complex structure. When applied to *Populus* microarray analysis, CorSig outperformed Zhu's for identification of co-expressed genes in the flavonoid biosynthetic pathway. We also showed that *p*-values obtained by CorSig can be directly used as a parameter to depict the strength of co-expression in a concentric stratification graph (see Fig. 5), in lieu of a significance cutoff, to facilitate an integrative interpretation of biological and statistical significance of the observed co-expression patterns.

Despite the usefulness of correlation analysis, there are known limitations. For instance, performing correlation analysis with small sample sizes is generally not advisable. PCC, in particular, is susceptible to the influence of outliers or data noise [50], and insensitive to non-linear relationships [51]. As demonstrated for the *Populus* dataset, CorSig is not correlation method-dependent, and is extendable to other algorithms, such as the Spearman correlation (Fig. 6E–H). We envision its application to other correlation measures as well, such as mutual information [14] and maximum information coefficient [52]. CorSig is simple to implement and effective for multiple data scenarios. As such, CorSig should be applicable to a wide range of applications, particularly for high-dimensional genomic data.
